# Cardiogenic shock and tumor resection due to cardiac synovial sarcoma: a case report

**DOI:** 10.1186/s43044-022-00293-7

**Published:** 2022-07-15

**Authors:** Ingeborg M. Keeling, Manuela A. Aschauer, Ameli E. Yates

**Affiliations:** 1 Department of Cardiac Surgery, Medical University Graz, Auenbruggerplatz 29, 8036 Graz, Austria; 2 Department of Radiology, Medical University Graz, Graz, Austria

**Keywords:** Cardiac, Monophasic synovial sarcoma, Genetics, Resection, Case report

## Abstract

**Background:**

Cardiac synovial sarcoma of the heart is a rare, aggressive mesenchymal tumor with poor prognosis, since complete resection is seldom feasible.

**Case presentation:**

A 23-year-old man was referred in cardiogenic shock. Emergency computed tomography (CT) revealed a large tumor with obstruction of the right atrium (RA) and prolapse into the right ventricle (RV). Resection and pericardial patch plasty were performed. Histology confirmed a G-3 spindle-cell sarcoma. At 21 months postoperatively, CT and cardiac magnetic resonance (MR) angiography showed a tumor emerging from the lateral wall of the superior caval vein (SCV) and the RA. The RA and SCV were completely resected and replaced with a tailored Dacron tunnel prosthesis. Histology confirmed R0 resection of a G-3 spindle-cell sarcoma. Reverse transcription-polymerase chain reaction (RT-PCR) confirmed a monophasic fibrous synovial sarcoma. Echocardiography upon discharge showed normal biventricular function. The heart was tumor-free upon PET-CT 24 months thereafter. A sudden progression with innumerable pulmonary nodules caused only minimal exertional dyspnea, and the patient received palliative monochemotherapy with ifosfamide. Thirty months after the first operation, he succumbed to hemorrhage from a brain metastasis.

**Conclusions:**

We report an unusually long postoperative period of 30 months in our patient after resection of a very large right atrial sarcoma. Early diagnosis, aggressive surgical treatment, adjunctive chemotherapy and radiotherapy affect survival. Systematic inclusion of patients in multicenter initiatives, including biobanking, is necessary. Better knowledge of genetic defects relevant to these cardiac tumors will promote accurate diagnoses and suggest novel and personalized gene-based therapies.

## Background

We encountered one patient with a synovial sarcoma of the right atrium among 100 consecutive surgically treated tumor patients during the past 30 years. Among these patients, 9% were surgically treated for a malignant cardiac tumor, the majority being angiosarcoma [1]. Synovial sarcoma is a very rare entity of cardiac sarcomas originating from multipotent mesenchymal stem cells and lacks synovial differentiation [2]. A minority of the patients may escape an unfavorable outcome after complete resection. Very rarely, VAD implantation or orthotopic heart transplantation may be performed [3, 4].

## Case presentation

**Fig. 1:**
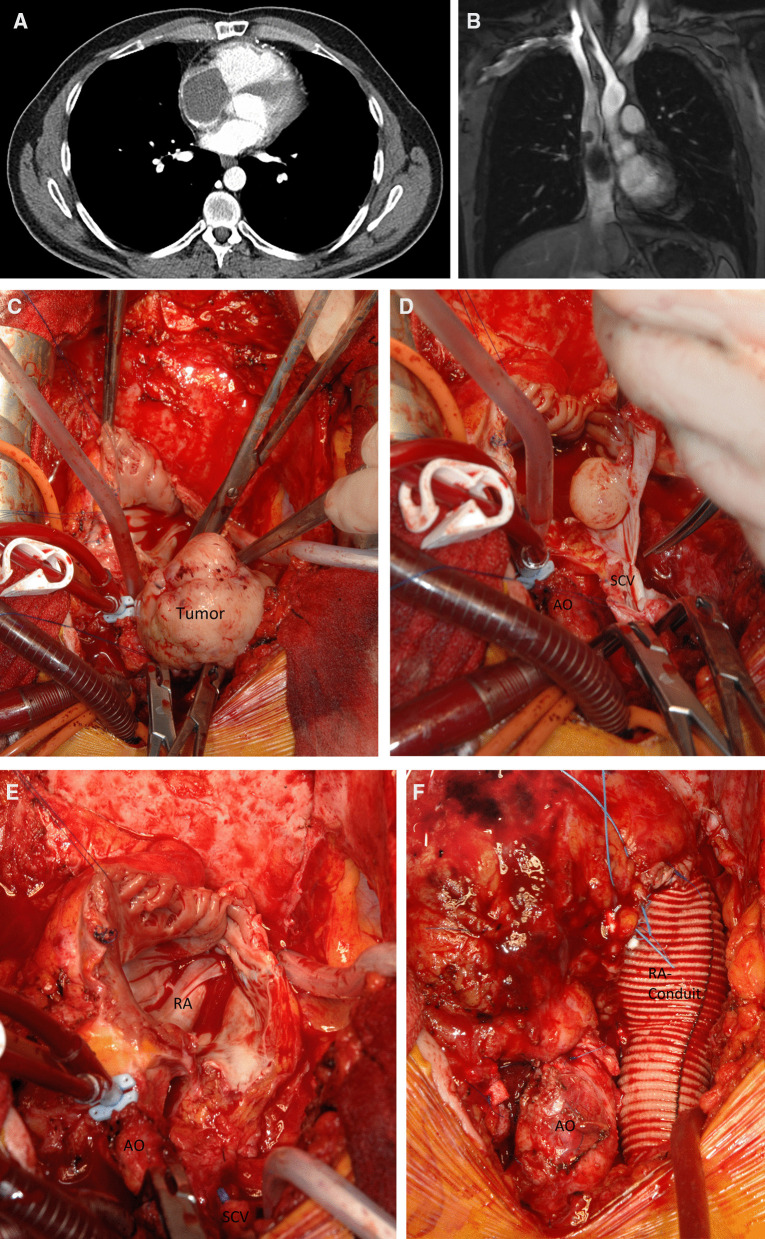
**A** CT shows a large RA tumor mass with subtotal obliteration of the tricuspid valve with a major portion prolapsing into the RV. **B** Cardiac MR angiography shows a large RA tumor mass emerging from the lateral wall of the SCV with subtotal obliteration of the RA. **C–E** Intraoperative view during complete resection of a RA tumor. **F** Intraoperative view after complete resection of a RA tumor and reconstruction with a tailored Dacron prosthesis

A 23-year-old man was referred to our department when transthoracic echocardiography (TTE) suggested a right atrial (RA) tumor. Emergency computed tomography (CT) revealed complete RA obstruction and tumor prolapse into the right ventricle (RV) (Fig. 1A), causing inflow congestion, pleural and minimal pericardial effusion. The patient, in cardiogenic shock, was immediately transferred to surgery. On moderately hypothermic non-beating heart bypass with snared caval veins, the firm tumor (8 × 5 × 5 cm, pedicle diameter 2.5 cm), adherent to the lateral RA wall, was macroscopically completely resected. The defect was closed with pericardial patch plasty. The NOS.G-3 spindle-cell sarcoma contained mitotic cells. Immunohistochemistry reacted positively with cytokeratin 7 and 19, EMA antibody. The proliferative ratio, evaluated with MIB1 (Ki-67), reached up to 60% of the cells. Fast-track extubation and perioperative course were uneventful. The patient was discharged with sinus rhythm and 65% ejection fraction (EF). Magnetic resonance (MR) imaging and positron emission tomography (PET) showed no metastases. Two cycles of radiotherapy (single dose of 1.8 Gy in the tumor region, with a tumor dose up to 50.4 Gy) were ordered when the final histology showed a few tumor cells near the resection margin.

At 21 months postoperatively, CT and cardiac MR angiography showed a tumor emerging from the lateral wall of the superior caval vein (SCV) and the RA (4.4 × 5.3 × 5.8 cm) (Fig. 1B). After careful resternotomy using an oscillating saw, meticulous preparation of massive adhesions after irradiation was performed. Moderately hypothermic non-beating heart bypass via the brachiocephalic vein and the right femoral vein was started. Both caval veins were snared. The ascending aorta was clamped, and 1000 ml of blood cardioplegia was applied. After incision of the right atrium, waste suction was used. The tumor, adherent to the wall of the superior caval vein, was resected in toto with the vessel wall to achieve clear margins. The complete free wall of the RA with the adherent tumor, the RA appendage, and the pericardial patch of the first operation were excised (Fig. 1C–E). After the cardiac tumor was completely resected, the wall defect was replaced with a tailored Dacron tunnel prosthesis from the superior caval vein sleeve to the inferior caval vein and the free wall of the RA (Fig. 1F). Histology confirmed R0 resection of a G-3 spindle-cell sarcoma. Tumor cells had 25 mitoses per 10 high power fields; distinct tissue necrosis was present. Immunohistochemistry was identical to the first tumor and S100 positive. Reverse transcription-polymerase chain reaction (RT-PCR) confirmed SYT/SSX1(t(X;18)(p11.2;q11.2)SYT/SSX) consistent with the specific diagnosis of a monophasic fibrous synovial sarcoma. TTE upon discharge showed normal biventricular function with EF 65%. The heart was tumor-free upon PET-CT 24 months thereafter. A sudden progression with innumerable pulmonary nodules caused only minimal exertional dyspnea, and the patient received monthly cycles of palliative monochemotherapy with ifosfamide, which he tolerated well. Thirty months after the first operation, he developed left-sided hemiparesis and succumbed to hemorrhage from a brain metastasis.

## Discussion

Reports on the postsurgical outcome with synovial sarcoma are scarce. Unfortunately, adjuvant treatment has not achieved long-term survival beyond a mean of 13.4 months (except for 1 patient with remarkable survival of 171 months) [2]. Repeat (up to 11 times) debulking seemed to improve survival and quality of life. Cardiac tumor resection eliminates the common symptom of dyspnea and may then improve the quality of life of a patient, even enabling a return to work, as wished by our patient. In general, it represents a chance to allow the patient a period with as much of his prior way of life as possible. Tumor location, especially at valves, primary size, resectability, and histological grade largely influence survival. Complete tumor resection is the standard of care and the most important determinant of local recurrence and length of disease-free survival [5]. In a small series of 60 well-documented synovial sarcoma patients, 9 had complete resection with tumor-free margins and 1 near-complete resection [2]. If complete resection is not feasible, (repeated) debulking and as much reduction of the cardiac tumor load as possible have an immediate effect on survival, functional class and quality of life, especially for patients with limited treatment options. Rarely, resection crossing anatomical borders of valves, coronary arteries, and conduction system, facilitated by an ex situ procedure or autotransplantation, can further improve survival [6]. Complete resection of sarcomas may allow survival beyond 3 years [7]. Resection of small (< 2.5 cm) non-valvular or (< 4.0 cm) pericardial synovial sarcomas allowed long-term survival (up to 171 months in one exceptional case), but postsurgical survival was only 12–15 months with larger (> 5 cm) intracardiac tumors [2]. Resection may, however, be limited if the tumor extends bilaterally into pulmonary arteries. Orthotopic heart and heart–lung transplantation provide the widest possible margins. Mean survival time may rarely exceed 2 years [4, 7], and an exceptional survival time of 8.5 years was recorded [4]. Implantation of a VAD system after tumor resection may be another rare option [3].

Tamponade and cardiogenic shock are typical features. Immediate life-saving tumor resection and drainage on cardiopulmonary bypass are indicated on first presentation of cancer-derived tamponade. A pericardio-pleural or pericardio-peritoneal fenestration is performed for recurrent chronic pericardial effusion.

Adjunctive chemotherapy and radiotherapy, as well as age, affect overall survival [2]. Awareness should also be raised of the recent identification of a systemic agent, regorafenib, associated with evidence of activity in synovial sarcoma (5.6 vs. 1.0 months of median progression-free survival vs. placebo in a randomized phase II study) [8]. Systematic inclusion of patients in multicenter initiatives, including biobanking, is necessary. Further identification of genetic defects relevant to cardiac tumors will not only promote accurate diagnosis, but also suggest novel and personalized gene-based therapies in the future.

Limitations associated with this case report may be those inherent in the case report genre [9]. However, our case offers an insight into the role of surgical treatment of a patient with very rare cardiac synovial sarcoma where individual reports are not frequently seen in the literature of these rare cases.

## Conclusions

Total tumor load determines outcome, and only surgery can immediately reduce it. Since life expectancy is twice as long for patients who undergo complete tumor resection, early diagnosis and aggressive surgical treatment have important prognostic and therapeutic implications.

## Data Availability

All data generated or analyzed during this study are included in this published article.

## Abbreviations

CT: Computed tomography
RA: Right atrium
RV: Right ventricle
MR: Magnetic resonance
SCV: Superior caval vein
RT-PCRf: Reverse transcription-polymerase chain reaction
PET: Positron emission tomography
VAD: Ventricular assist device
TTE: Transthoracic echocardiography
NOS.G-3: Not otherwise specified grade-3
EMA: Epithelial membrane antigen
MIB1 (Ki-67): Mouse monoclonal antibody identifying Ki-67
EF: Ejection fraction
Gy: Gray
SYT/SSX: SYT/SSX gene rearrangement
